# Supplementary material for: HIVprotI: an integrated web based platform for prediction and design of HIV proteins inhibitors
Source: J Cheminform. 2018 Mar 9;10:12. doi: 10.1186/s13321-018-0266-y (PMC5845081; doi:10.1186/s13321-018-0266-y)
Supplement: Supplementary file 2 — Additional file 2. Source code of HIVProtI web server. [file 13321_2018_266_MOESM2_ESM.zip › HIVprotI_Source-code/hivprotI/weka-3-6-6/doc/weka/gui/visualize/VisualizePanel.html]

VisualizePanel


---


|  |  |  |
| --- | --- | --- |
| **Overview** \| **Package** \| **Class** \| **Tree** \| **Deprecated** \| **Index** \| **Help** \| **Weka's home** | |  |
| **PREV CLASS**   **NEXT CLASS** | **FRAMES**    **NO FRAMES**     **All Classes** |  |
| SUMMARY: NESTED \| FIELD \| CONSTR \| METHOD | DETAIL: FIELD \| CONSTR \| METHOD |  |


---


## weka.gui.visualize Class VisualizePanel

```
java.lang.Object
  java.awt.Component
      java.awt.Container
          javax.swing.JComponent
              javax.swing.JPanel
                  weka.gui.visualize.PrintablePanel
                      weka.gui.visualize.VisualizePanel
```

**All Implemented Interfaces:**: java.awt.image.ImageObserver, java.awt.MenuContainer, java.io.Serializable, javax.accessibility.Accessible, PrintableHandler

**Direct Known Subclasses:**: ThresholdVisualizePanel

---

``` public class VisualizePanel extends PrintablePanel ```

This panel allows the user to visualize a dataset (and if provided) a
classifier's/clusterer's predictions in two dimensions.
If the user selects a nominal attribute as the colouring attribute then
each point is drawn in a colour that corresponds to the discrete value
of that attribute for the instance. If the user selects a numeric
attribute to colour on, then the points are coloured using a spectrum
ranging from blue to red (low values to high).
When a classifier's predictions are supplied they are plotted in one
of two ways (depending on whether the class is nominal or numeric).  
For nominal class: an error made by a classifier is plotted as a square
in the colour corresponding to the class it predicted.  
For numeric class: predictions are plotted as varying sized x's, where
the size of the x is related to the magnitude of the error.

**Version:**
:   $Revision: 7406 $

**Author:**
:   Mark Hall (mhall@cs.waikato.ac.nz), Malcolm Ware (mfw4@cs.waikato.ac.nz)

**See Also:**: Serialized Form

---

**Nested Class Summary**

**Nested classes/interfaces inherited from class javax.swing.JComponent**

`javax.swing.JComponent.AccessibleJComponent`

**Nested classes/interfaces inherited from class java.awt.Component**

`java.awt.Component.BaselineResizeBehavior`


**Field Summary**

**Fields inherited from class javax.swing.JComponent**

`TOOL_TIP_TEXT_KEY, UNDEFINED_CONDITION, WHEN_ANCESTOR_OF_FOCUSED_COMPONENT, WHEN_FOCUSED, WHEN_IN_FOCUSED_WINDOW`

**Fields inherited from class java.awt.Component**

`BOTTOM_ALIGNMENT, CENTER_ALIGNMENT, LEFT_ALIGNMENT, RIGHT_ALIGNMENT, TOP_ALIGNMENT`

**Fields inherited from interface java.awt.image.ImageObserver**

`ABORT, ALLBITS, ERROR, FRAMEBITS, HEIGHT, PROPERTIES, SOMEBITS, WIDTH`


**Constructor Summary**

`VisualizePanel()`
  
          Constructor

`VisualizePanel(VisualizePanelListener ls)`
  
          This constructor allows a VisualizePanelListener to be set.


| **Method Summary** | |
| --- | --- |
| `void` | `addActionListener(java.awt.event.ActionListener act)`             Add a listener for this visualize panel |
| `void` | `addPlot(PlotData2D newPlot)`             Set a new plot to the visualize panel |
| `int` | `getCIndex()`             Get the index of the attribute selected for coloring |
| `Instances` | `getInstances()`             Get the master plot's instances |
| `java.lang.String` | `getName()`             Returns the name associated with this plot. |
| `weka.gui.visualize.VisualizePanel.PlotPanel` | `getPlotPanel()`             Returns the underlying plot panel. |
| `boolean` | `getShowAttBars()`             Gets whether or not attribute bars are being displayed. |
| `boolean` | `getShowClassPanel()`             Gets whether or not the class panel is being displayed. |
| `int` | `getSIndex()`             Get the index of the shape selected for creating splits. |
| `int` | `getXIndex()`             Get the index of the attribute on the x axis |
| `int` | `getYIndex()`             Get the index of the attribute on the y axis |
| `static void` | `main(java.lang.String[] args)`             Main method for testing this class |
| `void` | `removeAllPlots()`             Removes all the plots. |
| `void` | `setColourIndex(int index)`             Sets the index used for colouring. |
| `void` | `setInstances(Instances inst)`             Tells the panel to use a new set of instances. |
| `void` | `setLog(Logger newLog)`             Sets the Logger to receive informational messages |
| `void` | `setMasterPlot(PlotData2D newPlot)`             Set the master plot for the visualize panel |
| `void` | `setName(java.lang.String plotName)`             Set a name for this plot |
| `void` | `setShapes(FastVector l)`             This will set the shapes for the instances. |
| `void` | `setShowAttBars(boolean sab)`             Set whether the attribute bars should be shown or not. |
| `void` | `setShowClassPanel(boolean scp)`             Set whether the class panel should be shown or not. |
| `void` | `setSIndex(int index)`             Set the shape for creating splits. |
| `void` | `setUpComboBoxes(Instances inst)`             initializes the comboboxes based on the data |
| `void` | `setXIndex(int index)`             Set the index of the attribute for the x axis |
| `void` | `setYIndex(int index)`             Set the index of the attribute for the y axis |

**Methods inherited from class weka.gui.visualize.PrintablePanel**

`getSaveDialogTitle, getWriter, getWriters, getXScale, getYScale, saveComponent, setSaveDialogTitle, setScale`

**Methods inherited from class javax.swing.JPanel**

`getAccessibleContext, getUI, getUIClassID, setUI, updateUI`

**Methods inherited from class javax.swing.JComponent**

`addAncestorListener, addNotify, addVetoableChangeListener, computeVisibleRect, contains, createToolTip, disable, enable, firePropertyChange, firePropertyChange, firePropertyChange, getActionForKeyStroke, getActionMap, getAlignmentX, getAlignmentY, getAncestorListeners, getAutoscrolls, getBaseline, getBaselineResizeBehavior, getBorder, getBounds, getClientProperty, getComponentPopupMenu, getConditionForKeyStroke, getDebugGraphicsOptions, getDefaultLocale, getFontMetrics, getGraphics, getHeight, getInheritsPopupMenu, getInputMap, getInputMap, getInputVerifier, getInsets, getInsets, getListeners, getLocation, getMaximumSize, getMinimumSize, getNextFocusableComponent, getPopupLocation, getPreferredSize, getRegisteredKeyStrokes, getRootPane, getSize, getToolTipLocation, getToolTipText, getToolTipText, getTopLevelAncestor, getTransferHandler, getVerifyInputWhenFocusTarget, getVetoableChangeListeners, getVisibleRect, getWidth, getX, getY, grabFocus, isDoubleBuffered, isLightweightComponent, isManagingFocus, isOpaque, isOptimizedDrawingEnabled, isPaintingForPrint, isPaintingTile, isRequestFocusEnabled, isValidateRoot, paint, paintImmediately, paintImmediately, print, printAll, putClientProperty, registerKeyboardAction, registerKeyboardAction, removeAncestorListener, removeNotify, removeVetoableChangeListener, repaint, repaint, requestDefaultFocus, requestFocus, requestFocus, requestFocusInWindow, resetKeyboardActions, reshape, revalidate, scrollRectToVisible, setActionMap, setAlignmentX, setAlignmentY, setAutoscrolls, setBackground, setBorder, setComponentPopupMenu, setDebugGraphicsOptions, setDefaultLocale, setDoubleBuffered, setEnabled, setFocusTraversalKeys, setFont, setForeground, setInheritsPopupMenu, setInputMap, setInputVerifier, setMaximumSize, setMinimumSize, setNextFocusableComponent, setOpaque, setPreferredSize, setRequestFocusEnabled, setToolTipText, setTransferHandler, setVerifyInputWhenFocusTarget, setVisible, unregisterKeyboardAction, update`

**Methods inherited from class java.awt.Container**

`add, add, add, add, add, addContainerListener, addPropertyChangeListener, addPropertyChangeListener, applyComponentOrientation, areFocusTraversalKeysSet, countComponents, deliverEvent, doLayout, findComponentAt, findComponentAt, getComponent, getComponentAt, getComponentAt, getComponentCount, getComponents, getComponentZOrder, getContainerListeners, getFocusTraversalKeys, getFocusTraversalPolicy, getLayout, getMousePosition, insets, invalidate, isAncestorOf, isFocusCycleRoot, isFocusCycleRoot, isFocusTraversalPolicyProvider, isFocusTraversalPolicySet, layout, list, list, locate, minimumSize, paintComponents, preferredSize, printComponents, remove, remove, removeAll, removeContainerListener, setComponentZOrder, setFocusCycleRoot, setFocusTraversalPolicy, setFocusTraversalPolicyProvider, setLayout, transferFocusBackward, transferFocusDownCycle, validate`

**Methods inherited from class java.awt.Component**

`action, add, addComponentListener, addFocusListener, addHierarchyBoundsListener, addHierarchyListener, addInputMethodListener, addKeyListener, addMouseListener, addMouseMotionListener, addMouseWheelListener, bounds, checkImage, checkImage, contains, createImage, createImage, createVolatileImage, createVolatileImage, dispatchEvent, enable, enableInputMethods, firePropertyChange, firePropertyChange, firePropertyChange, firePropertyChange, firePropertyChange, getBackground, getBounds, getColorModel, getComponentListeners, getComponentOrientation, getCursor, getDropTarget, getFocusCycleRootAncestor, getFocusListeners, getFocusTraversalKeysEnabled, getFont, getForeground, getGraphicsConfiguration, getHierarchyBoundsListeners, getHierarchyListeners, getIgnoreRepaint, getInputContext, getInputMethodListeners, getInputMethodRequests, getKeyListeners, getLocale, getLocation, getLocationOnScreen, getMouseListeners, getMouseMotionListeners, getMousePosition, getMouseWheelListeners, getParent, getPeer, getPropertyChangeListeners, getPropertyChangeListeners, getSize, getToolkit, getTreeLock, gotFocus, handleEvent, hasFocus, hide, imageUpdate, inside, isBackgroundSet, isCursorSet, isDisplayable, isEnabled, isFocusable, isFocusOwner, isFocusTraversable, isFontSet, isForegroundSet, isLightweight, isMaximumSizeSet, isMinimumSizeSet, isPreferredSizeSet, isShowing, isValid, isVisible, keyDown, keyUp, list, list, list, location, lostFocus, mouseDown, mouseDrag, mouseEnter, mouseExit, mouseMove, mouseUp, move, nextFocus, paintAll, postEvent, prepareImage, prepareImage, remove, removeComponentListener, removeFocusListener, removeHierarchyBoundsListener, removeHierarchyListener, removeInputMethodListener, removeKeyListener, removeMouseListener, removeMouseMotionListener, removeMouseWheelListener, removePropertyChangeListener, removePropertyChangeListener, repaint, repaint, repaint, resize, resize, setBounds, setBounds, setComponentOrientation, setCursor, setDropTarget, setFocusable, setFocusTraversalKeysEnabled, setIgnoreRepaint, setLocale, setLocation, setLocation, setSize, setSize, show, show, size, toString, transferFocus, transferFocusUpCycle`

**Methods inherited from class java.lang.Object**

`equals, getClass, hashCode, notify, notifyAll, wait, wait, wait`

**Constructor Detail**

### VisualizePanel

```
public VisualizePanel(VisualizePanelListener ls)
```

:   This constructor allows a VisualizePanelListener to be set.

    **Parameters:**: `ls` - the listener to use

---


### VisualizePanel

```
public VisualizePanel()
```

:   Constructor


**Method Detail**

### setLog

```
public void setLog(Logger newLog)
```

:   Sets the Logger to receive informational messages

    :   **Parameters:**: `newLog` - the Logger that will now get info messages

---


### setShowAttBars

```
public void setShowAttBars(boolean sab)
```

:   Set whether the attribute bars should be shown or not.
    If turned off via this method then any setting in the
    properties file (if exists) is ignored.

    :   **Parameters:**: `sab` - false if attribute bars are not to be displayed.

---


### getShowAttBars

```
public boolean getShowAttBars()
```

:   Gets whether or not attribute bars are being displayed.

    :   **Returns:**: true if attribute bars are being displayed.

---


### setShowClassPanel

```
public void setShowClassPanel(boolean scp)
```

:   Set whether the class panel should be shown or not.

    :   **Parameters:**: `scp` - false if class panel is not to be displayed

---


### getShowClassPanel

```
public boolean getShowClassPanel()
```

:   Gets whether or not the class panel is being displayed.

    :   **Returns:**: true if the class panel is being displayed.

---


### setColourIndex

```
public void setColourIndex(int index)
```

:   Sets the index used for colouring. If this method is called then
    the supplied index is used and the combo box for selecting colouring
    attribute is disabled.

    :   **Parameters:**: `index` - the index of the attribute to use for colouring

---


### setXIndex

```
public void setXIndex(int index)
               throws java.lang.Exception
```

:   Set the index of the attribute for the x axis

    :   **Parameters:**: `index` - the index for the x axis **Throws:**: `java.lang.Exception` - if index is out of range.

---


### getXIndex

```
public int getXIndex()
```

:   Get the index of the attribute on the x axis

    :   **Returns:**: the index of the attribute on the x axis

---


### setYIndex

```
public void setYIndex(int index)
               throws java.lang.Exception
```

:   Set the index of the attribute for the y axis

    :   **Parameters:**: `index` - the index for the y axis **Throws:**: `java.lang.Exception` - if index is out of range.

---


### getYIndex

```
public int getYIndex()
```

:   Get the index of the attribute on the y axis

    :   **Returns:**: the index of the attribute on the x axis

---


### getCIndex

```
public int getCIndex()
```

:   Get the index of the attribute selected for coloring

    :   **Returns:**: the index of the attribute on the x axis

---


### getSIndex

```
public int getSIndex()
```

:   Get the index of the shape selected for creating splits.

    :   **Returns:**: The index of the shape.

---


### setSIndex

```
public void setSIndex(int index)
               throws java.lang.Exception
```

:   Set the shape for creating splits.

    :   **Parameters:**: `index` - The index of the shape. **Throws:**: `java.lang.Exception` - if index is out of range.

---


### addActionListener

```
public void addActionListener(java.awt.event.ActionListener act)
```

:   Add a listener for this visualize panel

    :   **Parameters:**: `act` - an ActionListener

---


### setName

```
public void setName(java.lang.String plotName)
```

:   Set a name for this plot

    :   **Overrides:**: `setName` in class `java.awt.Component`
    :   **Parameters:**: `plotName` - the name for the plot

---


### getName

```
public java.lang.String getName()
```

:   Returns the name associated with this plot. "" is returned if no
    name is set.

    :   **Overrides:**: `getName` in class `java.awt.Component`
    :   **Returns:**: the name of the plot

---


### getInstances

```
public Instances getInstances()
```

:   Get the master plot's instances

    :   **Returns:**: the master plot's instances

---


### setShapes

```
public void setShapes(FastVector l)
```

:   This will set the shapes for the instances.

    :   **Parameters:**: `l` - A list of the shapes, providing that the objects in the lists are non editable the data will be kept intact.

---


### setInstances

```
public void setInstances(Instances inst)
```

:   Tells the panel to use a new set of instances.

    :   **Parameters:**: `inst` - a set of Instances

---


### setUpComboBoxes

```
public void setUpComboBoxes(Instances inst)
```

:   initializes the comboboxes based on the data

    :   **Parameters:**: `inst` - the data to base the combobox-setup on

---


### removeAllPlots

```
public void removeAllPlots()
```

:   Removes all the plots.

---


### setMasterPlot

```
public void setMasterPlot(PlotData2D newPlot)
                   throws java.lang.Exception
```

:   Set the master plot for the visualize panel

    :   **Parameters:**: `newPlot` - the new master plot **Throws:**: `java.lang.Exception` - if the master plot could not be set

---


### addPlot

```
public void addPlot(PlotData2D newPlot)
             throws java.lang.Exception
```

:   Set a new plot to the visualize panel

    :   **Parameters:**: `newPlot` - the new plot to add **Throws:**: `java.lang.Exception` - if the plot could not be added

---


### getPlotPanel

```
public weka.gui.visualize.VisualizePanel.PlotPanel getPlotPanel()
```

:   Returns the underlying plot panel.

    :   **Returns:**: the plot panel

---


### main

```
public static void main(java.lang.String[] args)
```

:   Main method for testing this class

    :   **Parameters:**: `args` - the commandline parameters


---


|  |  |  |
| --- | --- | --- |
| **Overview** \| **Package** \| **Class** \| **Tree** \| **Deprecated** \| **Index** \| **Help** \| **Weka's home** | |  |
| **PREV CLASS**   **NEXT CLASS** | **FRAMES**    **NO FRAMES**     **All Classes** |  |
| SUMMARY: NESTED \| FIELD \| CONSTR \| METHOD | DETAIL: FIELD \| CONSTR \| METHOD |  |


---
